# Automatic enhancement preprocessing for segmentation of low quality cell images

**DOI:** 10.1038/s41598-024-53411-7

**Published:** 2024-02-13

**Authors:** Sota Kato, Kazuhiro Hotta

**Affiliations:** 1 Department of Electrical, Information, Materials and Materials Engineering, Graduate School of Science and Engineering, Meijo University, Shiogamaguchi, Tempaku-ku, Nagoya, Aichi 468-8502 Japan; 2 Department of Electrical and Electronic Engineering, Faculty of Engineering, Meijo University, Nagoya, Aichi Japan

**Keywords:** Image processing, Machine learning

## Abstract

We present a novel automatic preprocessing and ensemble learning technique for the segmentation of low-quality cell images. Capturing cells subjected to intense light is challenging due to their vulnerability to light-induced cell death. Consequently, microscopic cell images tend to be of low quality and it causes low accuracy for semantic segmentation. This problem can not be satisfactorily solved by classical image preprocessing methods. Therefore, we propose a novel approach of automatic enhancement preprocessing (AEP), which translates an input image into images that are easy to recognize by deep learning. AEP is composed of two deep neural networks, and the penultimate feature maps of the first network are employed as filters to translate an input image with low quality into images that are easily classified by deep learning. Additionally, we propose an automatic weighted ensemble learning (AWEL), which combines the multiple segmentation results. Since the second network predicts segmentation results corresponding to each translated input image, multiple segmentation results can be aggregated by automatically determining suitable weights. Experiments on two types of cell image segmentation confirmed that AEP can translate low-quality cell images into images that are easy to segment and that segmentation accuracy improves using AWEL.

## Introduction

In recent years, segmentation tasks that assign class labels to each pixel in an image have become important in the field of medical and biological images^1–6^. Cell image segmentation is subjective because it has been performed manually; however, deep learning can obtain objective results. Many segmentation methods have been proposed for medical and biological images^7–15^, and further, it is more diverse and suitable for real-world environments is attracting attention such as instance segmentation^16^, 3D segmentation^17,18^, segmentation and tracking^19^, few-shot segmentation^20^, semi-supervised segmentation^21^, and lightweight model for segmentation^22^.

However, low quality image, which is the serious problem with real-world dataset in cell biology, has not been discussed. The segmentation accuracy of deep learning methods depends on the quality of the input images. In particular, cell images are of low quality, because cells die under strong light. To achieve high accuracy in cell image segmentation, appropriate image preprocessing is required for a deep learning model to easily understand the given input.

Moreover, few studies have focused on preprocessing suitable for deep learning. Typically, classical preprocessing methods, such as a Gaussian filter^23^ and bilateral filter^24^, are used. Although these methods can remove noise from images, the quality of the preprocessed images depends on hyperparameters, and their suitability for deep learning is difficult to conclude. Alternatively, in terms of clarifying the images, many image super-resolution methods have been proposed^25–32^. These methods require high-quality teacher images, and preparing these images requires considerable time and computational cost. In addition, preparing high-quality images for cell images is difficult because cells die under strong light.

**Figure 1:**
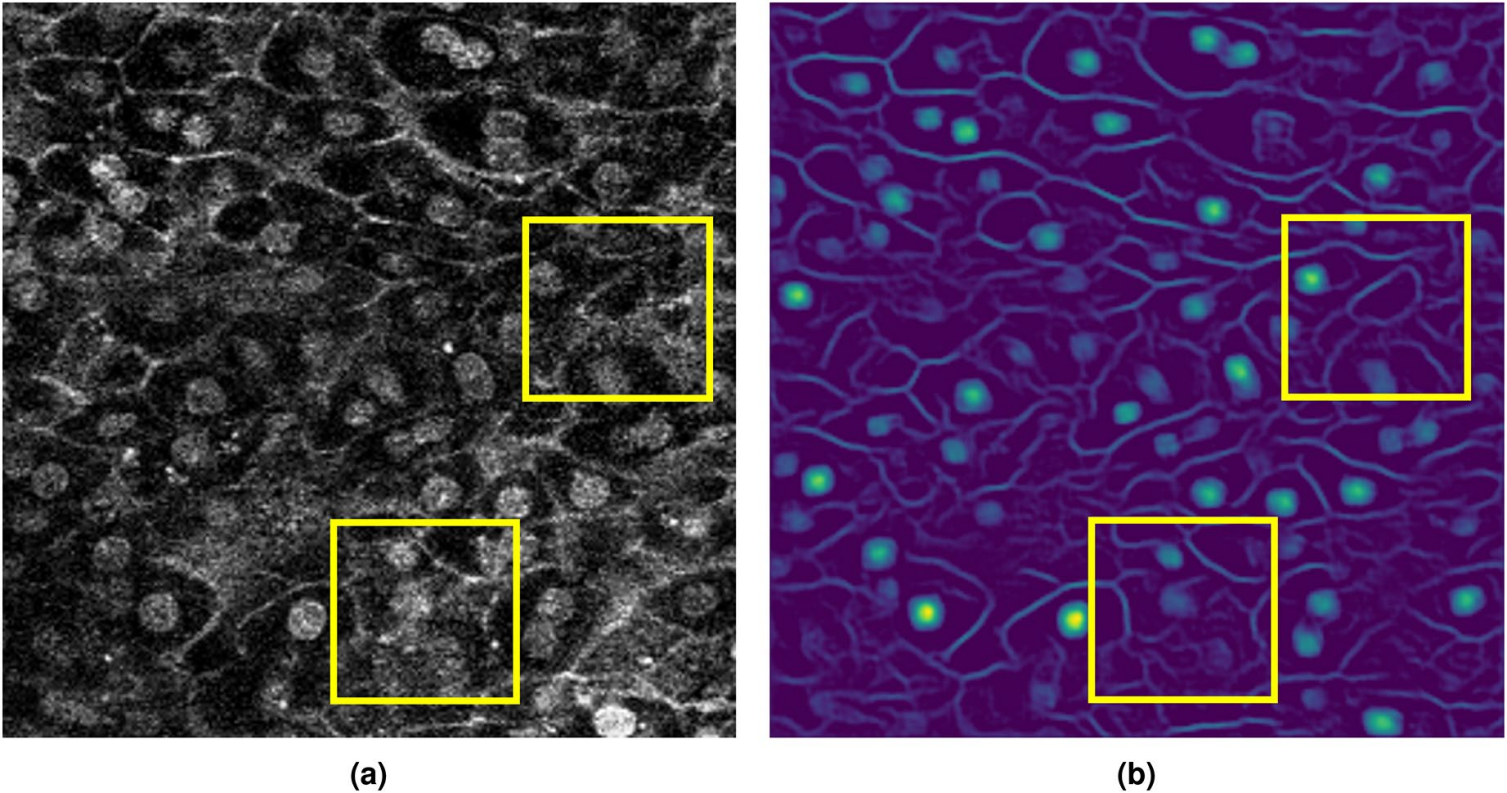
Examples of cell image and its penultimate feature map. (**a**) Low-quality cell image as input. (**b**) One of the penultimate feature maps when the cell image is fed to a model based on a CNN.

Therefore, we present a novel automatic pre-processing method for cell image segmentation using deep learning. Figure 1 presents examples of a low-quality cell image and penultimate feature map when the cell image is input into a model based on a convolutional neural network (CNN) trained on a cell image segmentation dataset. As shown in the yellow frame in Fig. 1, the penultimate feature map can capture cell membranes and nuclei that are not clear in the low-quality image. This result shows that the feature map of the CNN contains useful information for segmentation that is difficult to observe in low-quality images. Based on this analysis, we present a novel preprocessing method called automatic enhancement preprocessing (AEP).

**Figure 2:**
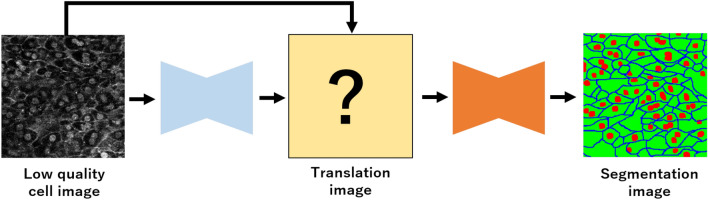
Overview of AEP. AEP consists of two deep neural networks. The first network preprocesses images, and the second network segments the images generated by the first network.

Figure 2 shows an overview of AEP. AEP consists of two deep neural networks. The first network is used for semantic segmentation, and the penultimate feature maps in the first network are used as filters to translate an input image into images that are easy to segment. The number of channels for the penultimate feature maps is the same as that for the segmentation classes, and the input cell image is translated into multiple images that emphasize each class. The second network is used to segment the images generated by the first network. The low-quality input cell image is translated by the filter, and the translated image was fed to the second network for segmentation.

Furthermore, we present automatic weighted ensemble learning (AWEL) to aggregate multiple segmented images generated by the first and second networks. Using AWEL, suitable weights are automatically determined, and the segmentation accuracy is further improved.

We conducted experiments to evaluate the proposed methods on two cell-segmentation datasets that distinguish cell images into multiple categories. The results confirmed that AEP can translate low-quality cell images into images that are easy to segment and that the segmentation accuracy improved using AWEL. Furthermore, add to^33^, we compared AEP with various previous network architectures^15,34^ and conventional preprocessing methods^23,24,35,36^, and analyzed AEP and AWEL architectures, which are the effectiveness of AWEL, the number of translation filters, and the difference of output between the first network and the second network, to confirm their effectiveness.

The remainder of this paper is organized as follows. Section "Related work" presents related work. Section "Method" explains the proposed method in detail. Section "Experiments" presents the experiment results. Finally, we summarize the study and discuss future work in Section "Discussion".

The main contributions of this study are summarized as follows:

- We present a novel automatic preprocessing method called AEP. The penultimate feature maps in the first network are used as filters to translate an input image into multiple images that emphasize each class, and the translated image is fed to the second network for semantic segmentation. We can obtain high-quality segmentation results using AEP even with low-quality input.
- Furthermore, we present AWEL to aggregate multiple segmentation results to determine suitable weights automatically. Consequently, the accuracy can be improved better than the general ensemble learning.

## Related work

### Biological image segmentation

In cell biology, semantic segmentation is a crucial task because segmentation results must be easy for humans to understand^37–39^, and deep learning methods have been widely spread^20,40^ because it can achieve higher accuracy. Further, in recent studies, it is more diverse and suitable for real environments is attracting attention such as instance segmentation^16^, 3D segmentation^17,18^, segmentation and tracking^19^, few-shot segmentation^20^, semi-supervised segmentation^21^, and lightweight model for segmentation^22^.

Recently, U-Net^10^ structure is a well-known segmentation method used in cell biology and medical image processing. It is an encoder-decoder network, and in the encoder, the features of an input image are extracted by convolution. Fine information, such as the correct object position, is lost during downsampling. In the decoder, skip connections are introduced at each resolution. Skip connections concatenate the feature maps obtained by the encoder with those of the same resolution in the decoder. Consequently, the fine information and correct positions lost during feature extraction can be used effectively. Furthermore, many network structures based on U-Net for improving accuracy have been proposed^11,12,14,15,41^. Li et al.^11^ proposed UNet++, which consists of U-Net with varying depths and whose decoders are densely connected at the same resolution using redesigned skip pathways. UNet++ addresses two key challenges: the unknown depth of the optimal architecture and unnecessarily restrictive design of the skip connections. Li et al.^15^ proposed shape-attentive U-Net (SAUNet), which focuses on model interpretability and robustness. SAUNet attempts to address the aforementioned limitations using a secondary shape stream that captures rich shape-dependent information in parallel with a regular texture stream.

Although there are many studies focusing on real environments in the biological imaging, there is little study into low image quality, which is the biggest issue in real environment images. Many segmentation methods also have been proposed for improving network structures to achieve the highest accuracy. However, the accuracy of biological segmentation depends on the input image quality. Our approach specializes in segmenting low-quality cell images, and it can translate input images into images that a CNN can easily classify.

### Image preprocessing

Image preprocessing methods include resizing, cropping, and color correction. Noise reduction is widely used for low-quality images. The most classical method for noise reduction is filtering^23,24,35^. Gaussian and bilateral filters^23,24^ can blur low-quality images and reduce noise, and the Sobel filter^35^ can emphasize object boundaries. However, the optimal parameters of these classical filters must be adjusted manually, and these parameters are sometimes unsuitable for deep learning.

Super-resolution methods that use deep learning^25–32^ are conceptually similar to the proposed method. Ledig et al.^27^ proposed SRGAN, which is a generative adversarial network for image super-resolution. SRGAN recovered photorealistic textures from heavily downsampled images on public benchmarks and achieved impressive gains in perceptual quality. Zhan et al.^29^ proposed very deep residual channel attention networks (RCAN) for image super-resolution. RCAN achieved higher accuracy and visual improvements compared with state-of-the-art image super-resolution methods. However, these methods require high-quality teacher images whose preparation is cost- and time-intensive. Recently, unsupervised super-resolution methods have been proposed^31,32^, but their image quality has been insufficient. Thus, using them to preprocess low-quality microscope images is difficult. GPU memory is also a problem because conventional networks for super-resolution enlarge the images.

Furthermore, a recent study proposed a learned image resizer using deep learning^42^. However, although this method is useful for image classification, it is ineffective for semantic segmentation.

Selecting a suitable preprocessing method is important for solving the actual cause of low-quality cell images. Unlike conventional methods, our proposed method can automatically preprocess cell images and simultaneously improve segmentation accuracy.

## Method

### Ethics

In our study, no patient-related images are taken during the experiments. For the mouse liver cell image dataset^43^, the animal protocols were reviewed and approved by the Animal Care and Use Committee of the Kyoto University Graduate School of Medicine (No. 10584), and all methods were performed in accordance with the guidelines and regulations.

### Automatic enhancement preprocessing (AEP)

**Figure 3:**
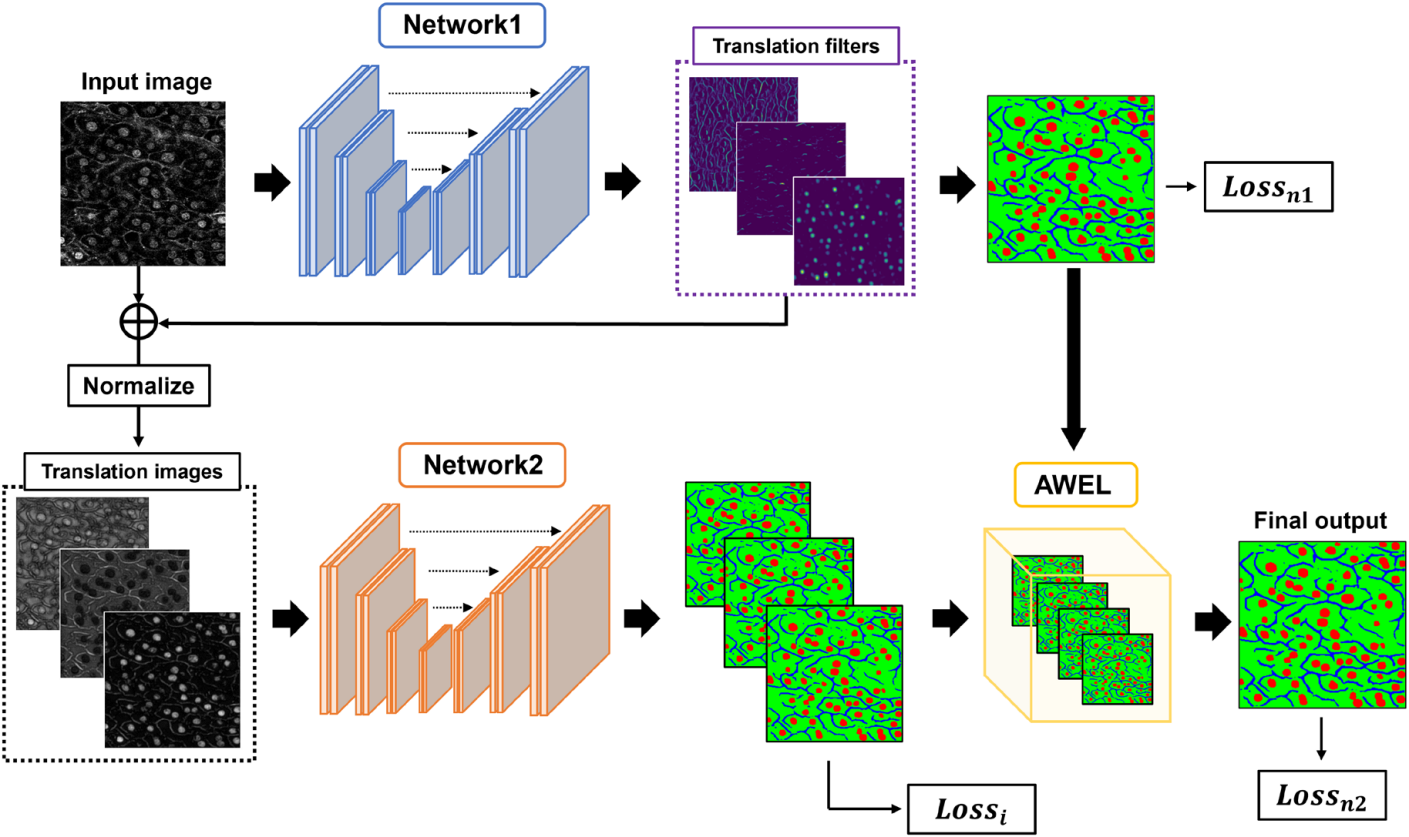
Overview of AEP+AWEL architecture. When we use a segmentation dataset of three classes, we set three translation filters. Each translation filter is added to an input cell image, and we obtain three translation images. Each translation image emphasizes objects related to the segmentation result. Translated images are fed to the second network one-by-one for segmentation, and we compute the loss for all segmentation images using AWEL.

We propose an unsupervised image translation method that uses deep learning to make an input image more suitable for segmentation. Figure 3 shows an overview of the proposed method. First, filters for translating input images into images suitable for segmentation are generated by the penultimate feature maps in the first network for cell image segmentation. The size of channels in the generated filters are the same as the input image. Because the first network outputs a segmentation image, the generated filters contain useful information for segmentation and emphasize objects related to the segmentation result. In this study, we call this is an automatic enhancement preprocessing using deep learning. We do not require high-quality ground truths to generate filters, and the method of generating filters for an input image to improve segmentation accuracy is also trained automatically.

When there are *N* datasets $(\{x_k,y_k\}_{k=1...N})$ of images $x_k$ and their labels $y_k$, we show the translation equation from the input image with low quality to translated images in Eq. (1).

1 $$\begin{aligned} \hat{x_{kc}} = x_k + Sigmoid(ReLU(f^{\prime }_1(x_k)_c) \end{aligned}$$

where $\hat{x}$ is the translated image, $f^{\prime }_1$ is the first network as the translation function, and *c* is the number of translation filters. The filters generated by penultimate feature maps of the first network are added to the input image $x_k$, and translated images $\hat{x_{kc}}$ that emphasize important regions are generated. However, if the filters contain negative values, the shapes of objects reflected in the input images may be erased. Therefore, we use the ReLU function before the filter output to avoid negative information in the filters. Finally, translated images are normalized from 0 to 1 using a sigmoid function because the luminance value is too large to interfere with learning. The generated filters are added to the input image, subsequently, and the translated images $\hat{x_{kc}}$ are fed to the second network $f_2$ for cell image segmentation. Because the number of translated images is the same as the number of translation filters, we feed each translated image to the second network $f_2$ independently, and the second network outputs multiple segmentation images. The segmentation results obtained from each translated image are different because each translated image differs from the original image. Finally, the segmented images generated by both the first network $f_1$ and the second network networks $f_2$ are aggregated using AWEL, and we generate the final segmentation image $z_k$ as shown in Eq. (2).

2 $$\begin{aligned} z_k = AWEL(f_1(x_{k}), f_2(\hat{x_{k1}}),..., f_2(\hat{x_{kc}})) \end{aligned}$$

We reduce the total error by aggregating the segmentation outputs. Both networks for filter generation and segmentation are simultaneously trained to generate highly accurate segmentation results.

For semantic segmentation, we use the softmax cross-entropy loss for all outputs in Eq. (3).

3 $$\begin{aligned} CE \, Loss = -\sum _{k=1}^N \sum _{c=1}^C y_{kc}\log p_{kc} \end{aligned}$$

where *C* is the number of categories in the dataset, $y_{kc}$ is the teacher label, and $p_{kc}$ is the probability value after a softmax function as $p_i=\frac{e^{z_i}}{\sum _{j} e^{z_j}}$. Further, $z_i$ is the *i*-th element of ${\textbf {z}}$, which is an output vector of the deep neural network. Equation (4) shows the final loss function.

4 $$\begin{aligned} Loss = CE \, Loss_{n1} + CE \, Loss_{n2} + \sum ^C_{c=1} CE \, Loss_{n3c} \end{aligned}$$

where $CE Loss_{n1}$ is the error of the first network output, $CE Loss_{n2}$ is the error of the outputs aggregated by AWEL, and $CE Loss_{n3c}$ is the error of the second network against the *c*-th translated image.

### Automatic weighted ensemble learning (AWEL)

The aim of ensemble learning is to aggregate the multiple segmentation images generated by the first and second networks into one segmentation result to improve segmentation accuracy. The ensemble has two types of averages: learning normal and weighted. In general, the weighted average is better if we assign large weights to important elements. However, determining suitable weight values is difficult. Therefore, we propose weighted ensemble learning, which automatically determines the weights using a 3D convolution layer.

**Figure 4:**
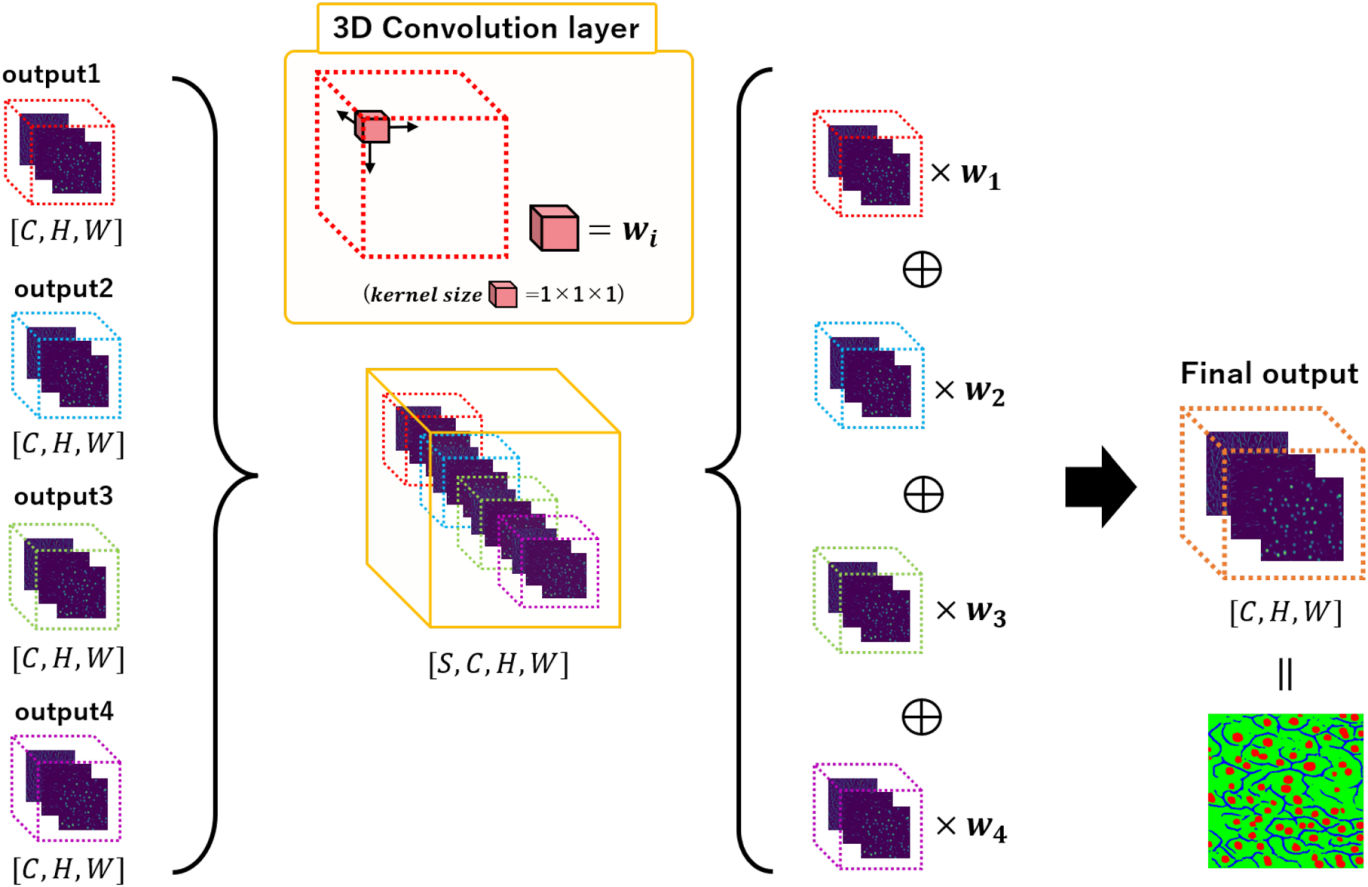
AWEL architecture using 3D convolution layer. In the segmentation of three classes, we prepare four segmentation outputs. The first network generates one segmentation result, and the second network generates three segmentation results. To aggregate all outputs and generate the final segmentation results, we use weighted ensemble learning. Weights are automatically determined by 3D convolution.

Figure 4 shows the architecture of the weighted ensemble learning. The shape of each segmentation result of the first and second networks is $[C \times H \times W]$, where *H* and *W* are the height and width of the output image, respectively, and *C* is the number of classes. All outputs are aggregated as $[S \times C \times H \times W]$, where *S* is the number of outputs. Here, we use a 3D convolution layer with $1\times 1\times 1$ kernels, a stride of 1, and padding of 0. This is called point-wise 3D convolution. Point-wise 3D convolution calculates only the channel direction. We can integrate this convolution layer into the aggregated array by replacing [*S*] in the aggregated array with the channel direction. Therefore, we can assign a weight $w_i$, as in Fig. 4, to each segmentation output $[C \times H \times W]$ through training, and automatically generate the final segmentation result from [*S*] results.

### Network structures

**Figure 5:**
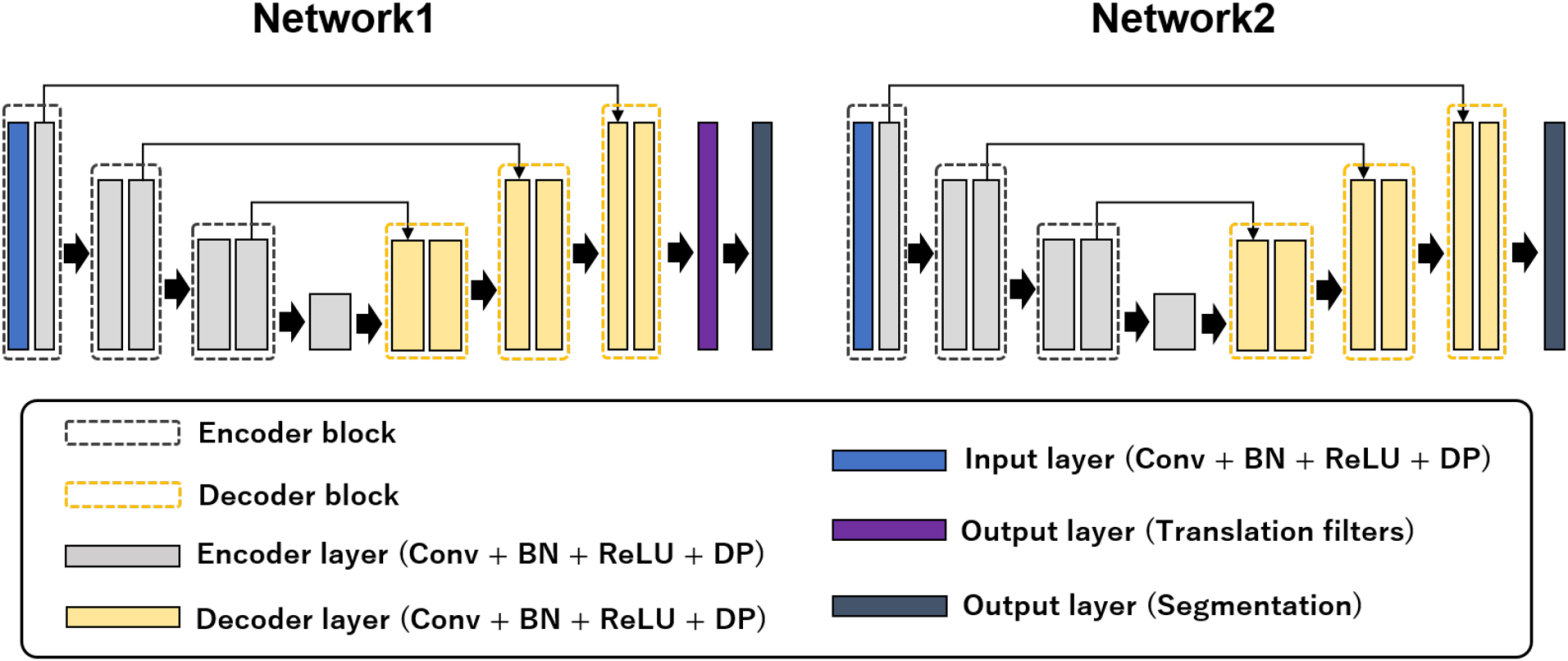
Overview of network structures. The proposed method is based on the U-Net architecture. The encoder and decoder networks consist of six layers. Each layer includes a convolution layer (Conv), batch normalization (BN), activation ReLU (ReLU), and dropout (DP). The first network (Network1) obtains segmentation results and translation filters using two output layers, and the second network (Network2) obtains segmentation results at the output layer.

Figure 5 shows an overview of the network structures. Our networks use encoder-decoder structures. We used a lighter structure than that of the original U-Net^10^ to reduce the number of calculations because we trained two types of networks simultaneously. As shown in Fig. 5, the encoder layer includes one convolution layer, batch normalization^44^, activation ReLU, and dropout^36^. The decoder layer includes a deconvolution layer, batch normalization, activation ReLU, and dropout. The encoder and decoder blocks consist of two encoders and two decoder layers, respectively. Although one encoder or decoder block consists of three convolution layers in the original U-Net, we remove convolution layers individually, including the encoder and decoder blocks, and the bottom-most block of the encoder consists of one encoder layer. The encoder network consists of one input layer and six encoder layers, and the decoder network consists of six decoder layers. Skip connections are introduced at each resolution.

In the first network, the output layer consists of two convolution layers. The outputs of the first convolution layer are used to translate an input image, and the output of the second convolution layer are used to predict each class. In the second network, only one convolution layer is used for semantic segmentation.

## Experiments

### Datasets

We used 50 cell images of a mouse liver with a ground truth attached by Kyoto University^43^. The ground truth image includes three labels: cytoplasm, nucleus, and membrane. The images and ground truth have a size of $256 \times 256$ pixels. Thirty-five images were used for training, five for validation, and the remaining 10 images for evaluation. We used 5-fold cross validation while replacing images for evaluation.

We also evaluated our method on another cell-image dataset. We used absorbance microscopy images of human iRPE cells (iRPE dataset)^13^. The ground truth includes two types of labels: background and membrane. The images were split into 1032 regions of $256 \times 256$ pixels and their corresponding ground truths. We randomly rearranged the images, divided each dataset into 2 to 1 in numerical order, and prepared them as training or inference data. We divided the inference data into validation and test data (1:2) and used 3-fold cross validation while switching the training and inference data.

Additionally, we used 2D electron microscopy images of the ISBI2012 challenge (ISBI2012)^45^ as a pseudo low quality dataset. This dataset is for binary segmentation of tubular structures spread over an image, i.e., cell membrane and background. We processed the original cell images in three ways to create three types of pseudo low quality cell images: (1) adding the random noise, (2) changing the contrast, and (3) adding the blur. For the random noise, we used the Gaussian noise ($\mu = 0$, $\sigma = 100$). For changing image contrast, we also used the Gaussian noise ($\mu = -100$, $\sigma = 0$), and we used a Gaussian filter (kernel size = 5) to add the blur. Since the resolution of ISBI2012 image is $512 \times 512$, we cropped a region of $256 \times 256$ pixels from the original images due to the limitation of GPU memory. There is no overlap for cropping areas, and consequently, the total number of crops is 120. We randomly rearranged the images. Afterward, we divided each dataset into 2 to 1 in index order and prepared them as training or inference data, and used 3-fold cross validation while switching the training and inference data.

**Figure 6:**
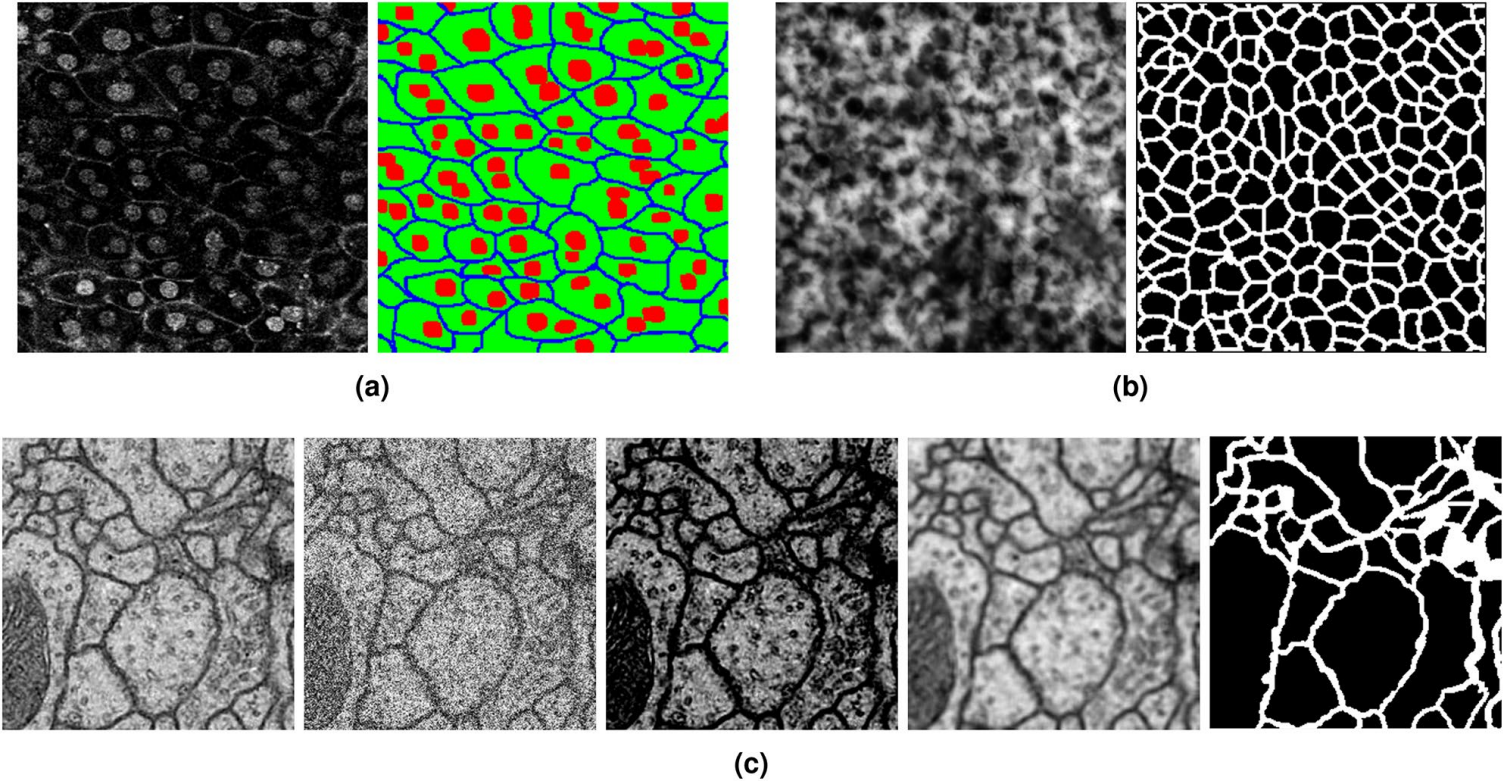
Examples of cell images and their ground truths in two datasets. (**a**) shows the cell image of a mouse liver with three class labels: cell nucleus (red), cell membrane (blue), and cytoplasm (green). (**b**) shows a human iRPE cell image labeled as: cell membrane (white) and background (black). (**c**) shows ISBI2012 dataset with pseudo low quality: cell membrane (white) and background (black).

Figure 6 shows examples of cell images in the three datasets and their ground truths. Figure 6a shows a mouse liver cell image and its ground truth with three classes: cell nucleus (red), cell membrane (blue), and cytoplasm (green). Figure 6b shows a human iRPE cell image with two class labels: cell membrane (white) and background (black), and Fig. 6c shows ISBI2012 dataset with pseudo low quality: cell membrane (white) and background (black).

### Training conditions and evaluation metrics

The images were normalized between 0 and 1, and no other preprocessing was performed. The batch size for training was set to 16, and Adam *(betas = 0.9, 0.999)* was used for optimization. The learning rate was set to $1 \times 10 ^ {-3}$. We trained all networks for 300 epochs, which is converged the training loss for all models and networks. The experiments evaluated AEP+AWEL and conventional segmentation networks^10,11,14,15,34^ without preprocessing to demonstrate the effectiveness of AEP and AWEL. Furthermore, we evaluate conventional image preprocessing methods based on filters^23,24,35,36,46^. All experiments were conducted using the same dataset size, optimizer, and number of epochs, and a single Nvidia GTX 1080Ti GPU was used as a calculator.

The segmentation accuracy of each class was evaluated using the interactive over union (IoU) and Dice score coefficient (DSC). The IoU and DSC compute the overlapping ratio between the predicted result and ground truth. Because the number of pixels in each class was different, we used the average score as the final evaluation measure.

### Results on cell image with low quality

#### Comparison with conventional models

Table 1 shows the segmentation results for the mouse liver cell image dataset. We evaluated the conventional methods^10,11,14,15,34^ and AEP+AWEL. The AEP+AWEL method improved the IoU by approximately 1.41% for cell nuclei and 2.95% for cell membranes compared with U-Net without preprocessing. The DSC of our method improved by approximately 1.04% for cell nuclei and 3.00% for cell membranes. The average IoU improved by approximately 1.63%, and the average DSC by approximately 1.48%. Surprisingly, the ground truth was not used for translated images, but adequate preprocessing for segmentation was realized. This result demonstrates the effectiveness of the proposed automatic preprocessing method.

**Table 1:** Comparison between the conventional and proposed methods on the cell image dataset of mouse livers.

Methods	IoU (%)				DSC (%)			
	Average	Cytoplasm	Membrane	Nucleus	Average	Cytoplasm	Membrane	Nucleus
U-Net	57.90±1.33	71.38±4.80	40.32±3.84	62.01±2.65	72.36±0.73	83.21±3.31	57.36±3.95	76.52±2.01
U-Net++	57.29±1.23	71.38±4.67	39.80±4.20	60.68±3.16	71.84±0.59	83.21±3.22	56.82±4.35	75.48±2.42
Attention U-Net	58.06±1.21	71.98±3.83	40.00±3.49	62.19±2.79	72.46±0.69	83.65±2.58	57.06±3.64	76.65±2.08
U-Net+++	56.80±1.24	**72.60±3.77**	37.18±4.95	60.63±2.58	71.18±1.11	**84.07±2.54**	54.01±5.33	75.45±2.00
SAUNet	58.47±1.80	72.13±3.43	41.71±2.26	61.58±3.75	72.92±1.17	83.76±2.32	58.83±2.26	76.16±2.84
AEP+AWEL	**59.53±1.72**	71.91±3.91	**43.27±2.32**	**63.42±3.38**	**73.84±1.06**	83.60±2.64	**60.36±2.26**	**77.56±2.50**

Significant values are in bold.

We also evaluated the proposed method using cell membrane datasets. Table 2 shows the results for the human iRPE cell images. The AEP+AWEL method improved the IoU by approximately 2.55% and the DSC by approximately 2.24% for cell membranes. The average IoU improved by approximately 1.19% and the average DSC by approximately 1.07% compared with the baseline U-Net without preprocessing. The results demonstrate that the proposed method is effective for other cell-image datasets.

**Table 2:** Comparison between the conventional and proposed methods on human iRPE cell images.

Methods	IoU (%)			DSC (%)		
	Average	Background	Membrane	Average	Background	Membrane
U-Net	62.86±0.37	**76.09±0.09**	49.64±0.69	76.38±0.32	**86.42±0.06**	66.34±0.61
U-Net++	62.33±0.68	74.97±0.68	49.69±0.89	76.04±0.54	85.69±0.45	66.38±0.79
Attention U-Net	63.95±0.45	76.07±0.40	51.82±0.53	77.34±0.35	86.41±0.26	68.26±0.46
U-Net+++	62.82±0.34	74.96±0.08	50.68±0.61	76.48±0.29	85.69±0.05	67.27±0.53
SAUNet	63.81±0.37	76.08±0.23	51.54±0.56	77.22±0.30	86.41±0.15	68.02±0.48
AEP+AWEL	**64.05±0.45**	75.92±0.33	**52.19±0.75**	**77.45±0.37**	86.31±0.22	**68.58±0.65**

Significant values are in bold.

**Figure 7:**
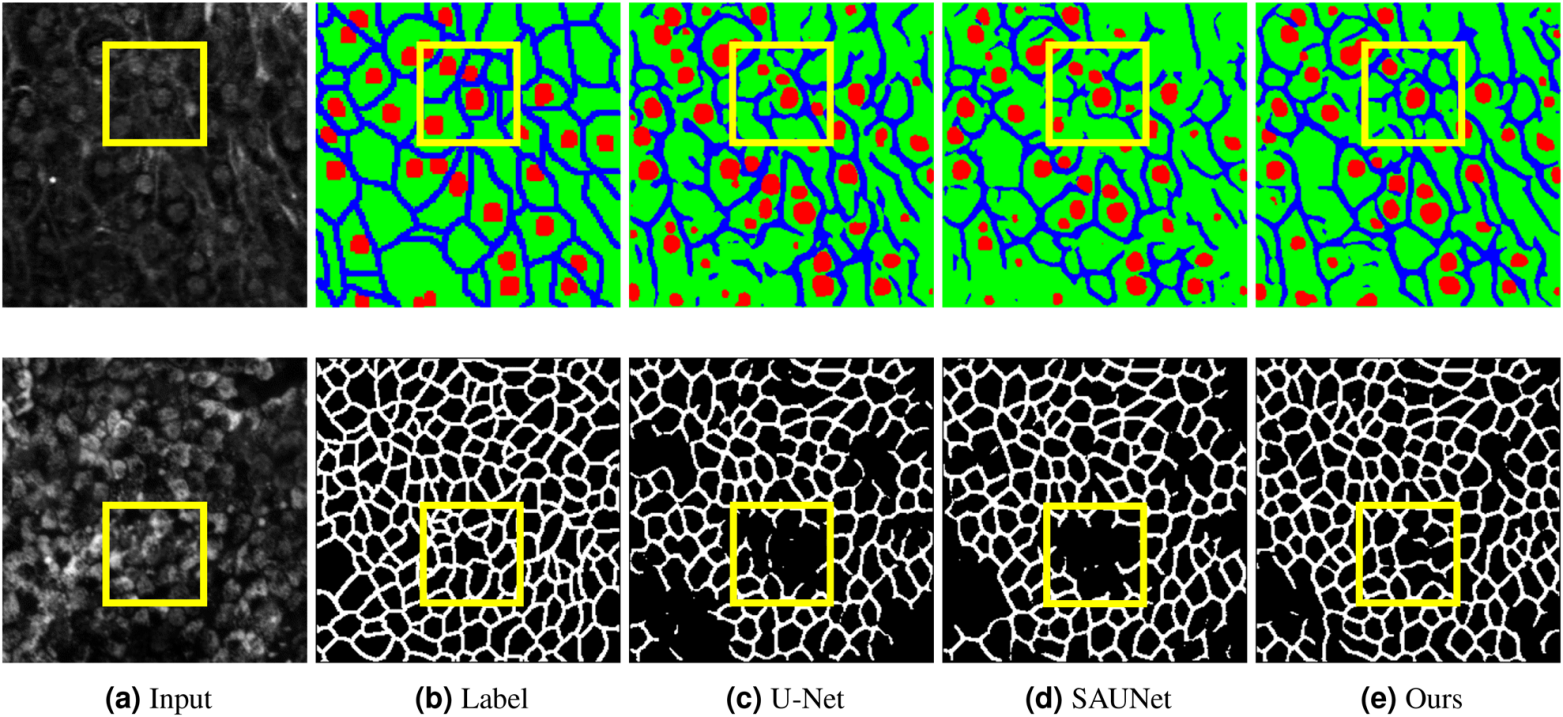
Qualitative results. (**a**) Input image, (**b**) label annotation, (**c**) U-Net, (**d**) SAUNet, and (**e**) AEP+AWEL (Ours).

Figure 7 visualizes the segmentation results for the two types of cell-image datasets. Focusing on the yellow squares in Fig. 7, the proposed method can segment cell membranes that conventional U-Net and SAUNet cannot segment well. Our method worked well even if the input images differed significantly from the previous experiment. The segmentation accuracy of the proposed method is better than those of U-Net and SAUNet without preprocessing.

#### Comparison with preprocessing methods

Table 3 shows the results of conventional image preprocessing methods. We evaluated the conventional filtering methods^23,24,35,36^ and our automatic preprocessing method. The kernel size of all filtering methods was set to $3 \times 3$ and $9 \times 9$. As shown in Table 3, AEP achieved the best accuracy for the two types of cell image datasets. For the mouse liver cell image dataset, although conventional preprocessing methods ineffectively improved the segmentation accuracy, AEP improved the IoU score of cell membranes and nuclei. On the iRPE cell image dataset, although the conventional filtering methods, except the bilateral filter, tended to reduce the accuracy, our preprocessing method achieved better IoUs in all classes.

**Table 3:** Comparison between conventional preprocessing methods.

Methods	Mouse liver cell image dataset				iRPE cell image dataset		
	Average	Cytoplasm	Membrane	Nucleus	Average	Background	Membrane
w/o preprocessing	57.90±1.33	71.38±4.80	40.32±3.84	62.01±2.65	62.86±0.37	**76.09±0.09**	49.64±0.69
Median filter (kernel size=3)	57.94±1.72	72.55±3.36	39.53±2.81	61.74±3.76	58.10±0.46	73.76±0.21	42.44±0.93
Median filter (kernel size=9)	50.01±2.29	67.59±5.25	32.15±4.22	50.29±4.56	39.38±0.16	62.43±1.18	16.33±1.30
Gaussian filter (kernel size=3)	57.62±1.17	71.79±4.07	40.18±3.95	60.90±1.72	59.50±0.23	74.67±0.18	44.33±0.32
Gaussian filter (kernel size=9)	56.32±1.80	71.12±3.96	38.81±2.62	59.04±3.91	49.80±1.26	69.10±0.74	30.50±3.18
Bilateral filter (kernel size=3)	58.38±1.40	**72.60±3.90**	40.66±3.47	61.86±2.79	62.88±0.39	75.81±0.31	49.95±0.49
Bilateral filter (kernel size=9)	58.41±1.29	72.22±4.34	40.75±4.10	62.90±2.49	62.90±0.39	76.05±0.06	49.74±0.76
Sobel filter (kernel size=3)	55.13±1.57	70.18±4.42	37.62±2.67	57.59±2.65	50.22±0.64	72.42±0.12	28.02±1.39
Sobel filter (kernel size=9)	55.84±1.95	70.04±3.52	38.62±2.77	58.87±3.05	34.93±0.12	69.86±0.25	0.00±0.00
Frangi/vesselness filter	53.09±0.18	69.43±1.38	32.59±2.41	57.24±3.79	55.27±2.29	72.83±0.93	37.71±2.76
AEP	**59.27±1.81**	72.40±3.92	**42.44±2.27**	**62.98±3.57**	**63.81±0.39**	75.98±0.32	**51.64±0.71**

Significant values are in bold.

**Figure 8:**
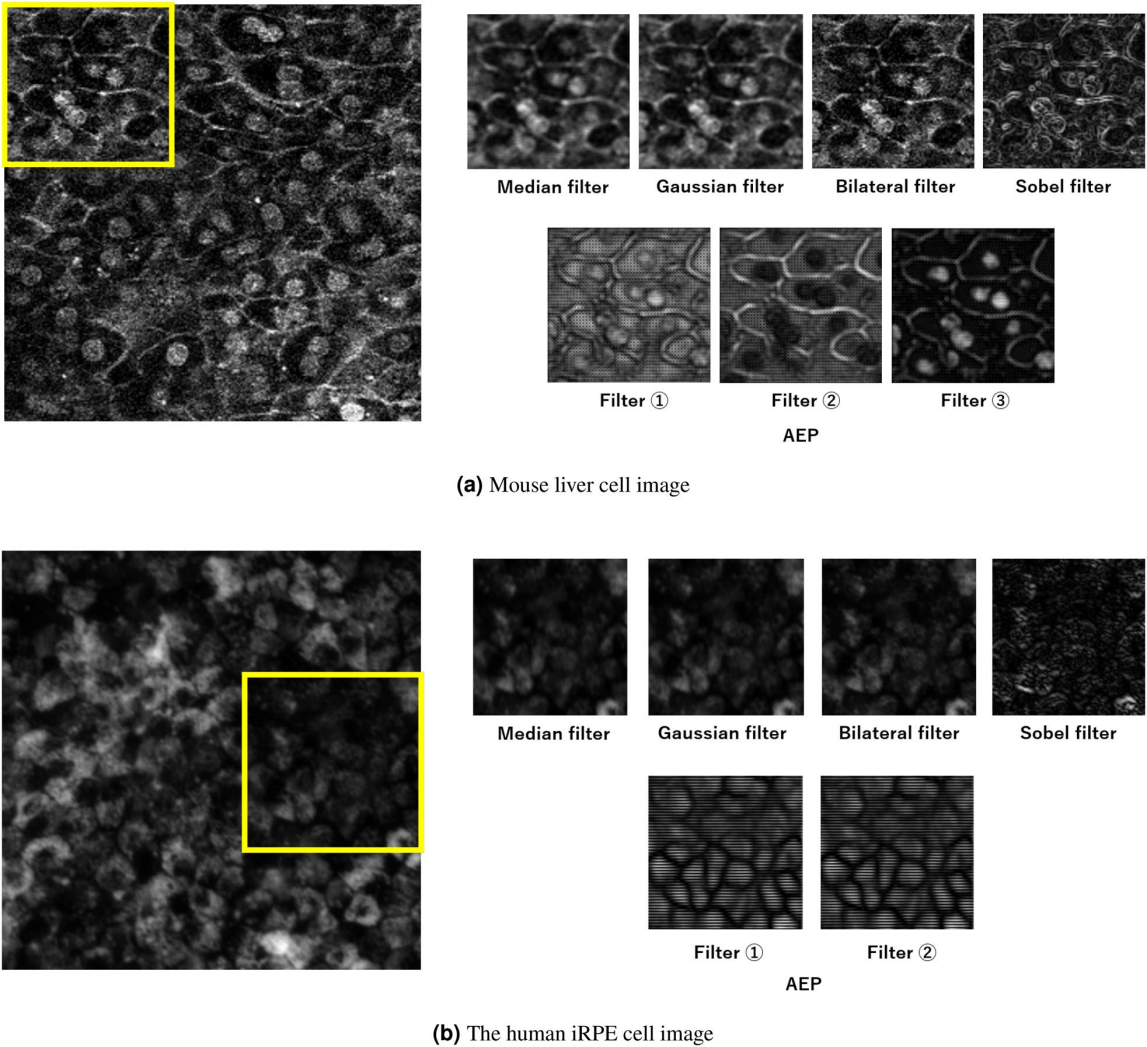
Visualization results of image preprocessing.

Figure 8 visualizes the results of image preprocessing. Focusing on the yellow squares in Fig. 8, for the mouse liver cell image dataset, confirming cell nuclei and membranes with low brightness in the original image was impossible. When the median and Gaussian filters were used, noise in the original image was reduced, but the output images were blurred. The bilateral filter was nearly unchanged in terms of quality, and the Sobel filter emphasized the edges of the object too much and consequently retained its shape as a cell. However, using AEP, cell nuclei with low brightness became clear, and cell membranes, which had become similar to noise, were more clearly emphasized. Although the cell membranes in the noisy part were difficult for humans to segment, we confirmed that the cell membrane is emphasized more by the filter, and the generated filter is suitable for segmentation. The IoU on the cell membranes using AEP improved by 2.62%. For the iRPE cell image dataset, although the conventional filtering methods were minimally effective, AEP generated a preprocessing image that emphasized the cells. These results demonstrate the effectiveness of our translation filter in that the necessary information for segmentation in the image is emphasized, and unnecessary information is suppressed.

### Results on cell image with pseudo low quality

Table 4 shows the segmentation results for ISBI2012 dataset with pseudo low quality. In Table 4, “Noise” means adding Gaussian noise, “Contrast” means changed the image contrast, and “Blur” means used the Gaussian filter for the input image to blur. We evaluated the baseline model (U-Net) and our AEP+AWEL using the IoU metric. As shown in Table 4, AEP+AWEL improved the IoU by approximately over 1.00% for cell membrane compared with U-Net. Consequently, the average IoU improved by approximately 1.66% for the noise, by approximately 1.74% for the contrast, and by approximately 1.82% for the blur. We believe that these results demonstrate the generalization performance of AEP+AWEL.

**Table 4:** Comparison between the conventional and proposed methods on the cell image datasets with pseudo low quality.

Methods	Noise			Contrast			Blur		
	Average	Background	Membrane	Average	Background	Membrane	Average	Background	Membrane
U-Net	76.34±0.76	88.26±0.34	64.42±1.21	79.24±0.76	89.76±0.43	68.71±1.10	79.40±0.51	89.95±0.29	68.85±0.76
AEP+AWEL	**78.00±1.02**	**88.79±0.54**	**66.21±1.52**	**80.98±0.72**	**90.23±0.38**	**70.74±1.06**	**81.22±0.72**	**90.38±0.37**	**71.06±1.08**

Significant values are in bold.

**Figure 9:**
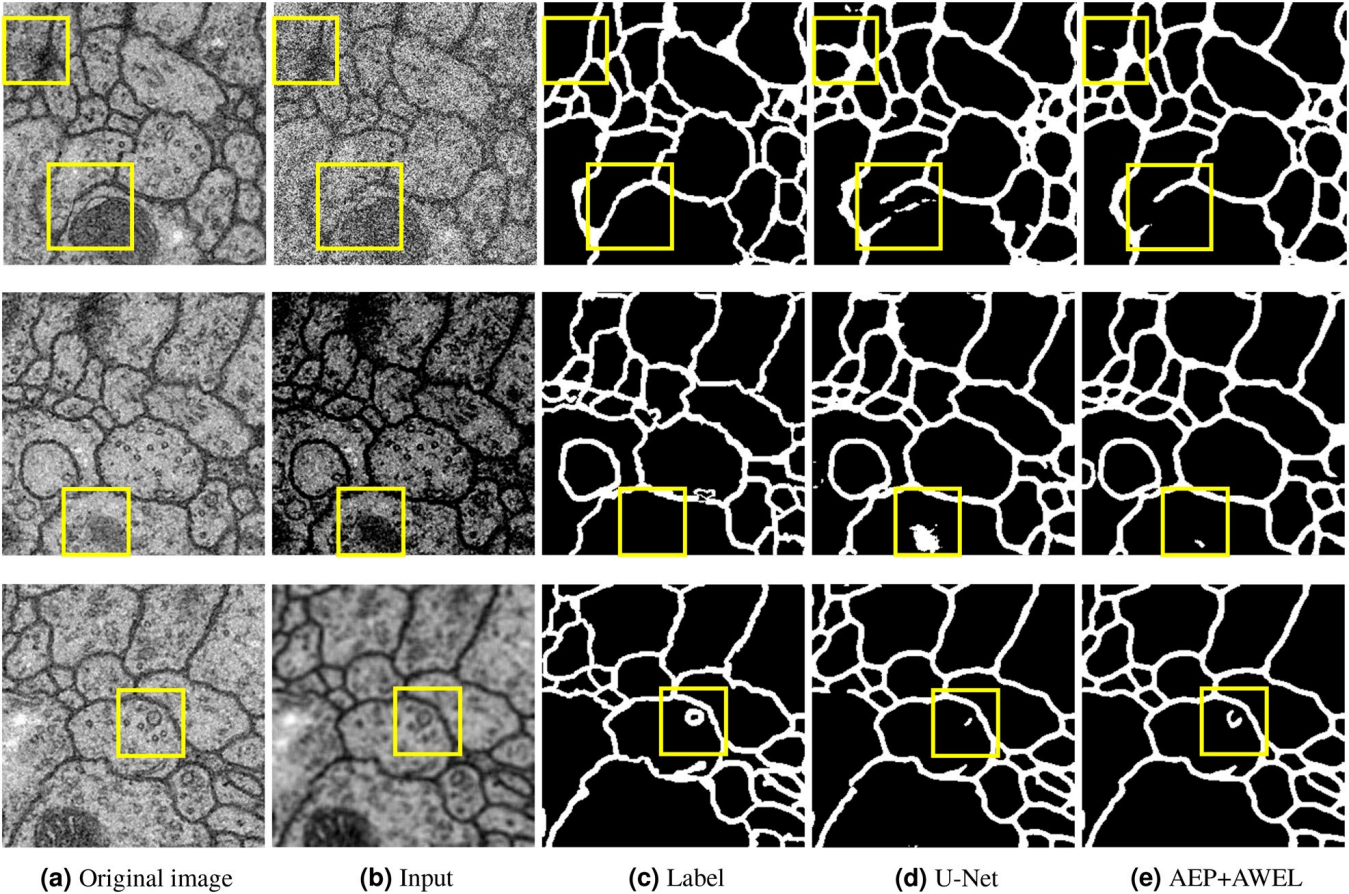
Qualitative results. (**a**) Original input image, (**b**) Input image with pseudo low quality (**c**) label annotation, (**d**) U-Net, and (**e**) AEP+AWEL (Ours).

Figure 9 visualizes the segmentation results for ISBI2012 dataset with pseudo low quality. Focusing on the yellow squares in Fig. 8, there are some miss-predictions regions in what is originally the background class as a result of pseudo-degradation. However, by using AEP+AWEL, we can be to control over-detection, and get a more accurate segmentation result. We confirmed that the generalization performance of our proposed preprocessing method from a qualitative aspect as well.

### Ablation studies

#### Effectiveness of AEP

Table 5 shows the results of the ablation studies for AEP. We compared our proposed AEP+AWEL with AEP used outputs of the first network instead of penultimate feature maps to confirm whether the penultimate feature maps are the most effective for preprocessing. Furthermore, we also evaluated outputs used the softmax layer and the argmax layer. As shown in Table 5, our proposed translation method used the penultimate feature maps was the best average IoU, and we consider that this is because the penultimate feature maps can get more detailed information as shown in Figure 1. We confirmed that the penultimate feature maps were more effective than feature maps of outputs.

**Table 5:** Ablation study for ensemble learning.

Methods	Mouse liver cell image dataset				iRPE cell image dataset		
	Average	Cytoplasm	Membrane	Nucleus	Average	Background	Membrane
AEP	59.27±1.81	**72.40±3.92**	42.44±2.27	62.98±3.57	63.81±0.39	**75.98±0.32**	51.64±0.71
AEP+AWEL (fixed)	59.41±0.73	72.01±0.73	42.94±0.73	63.26±0.73	63.88±0.48	75.71±0.28	52.06±0.69
AEP+AWEL (automated)	**59.53±1.72**	71.91±3.91	**43.27±2.32**	**63.42±3.38**	**64.05±0.45**	75.92±0.33	**52.19±0.75**

Significant values are in bold.

**Figure 10:**
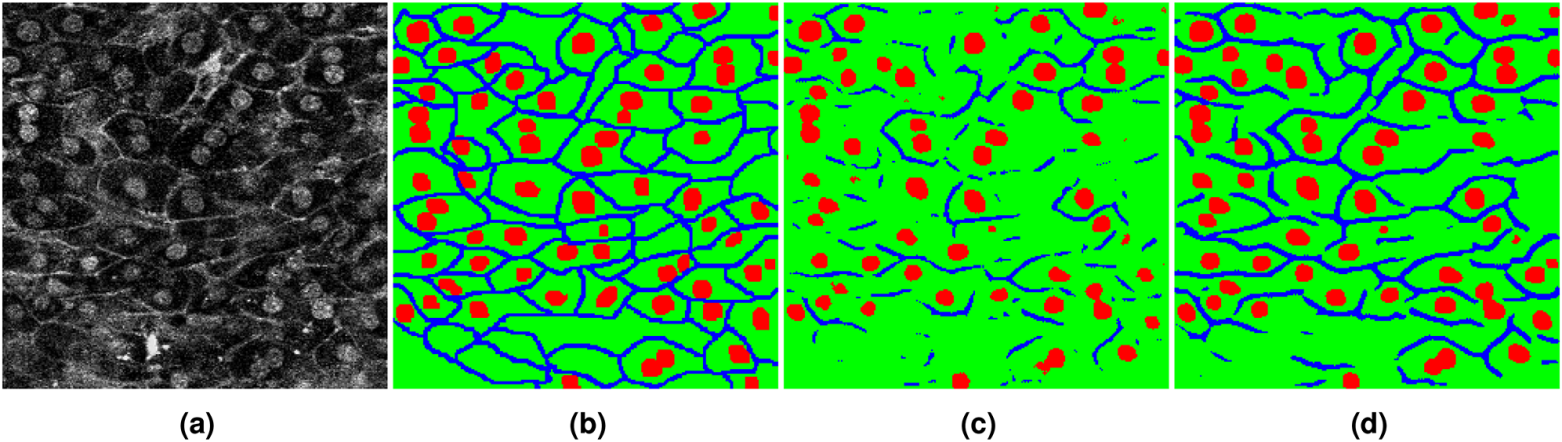
Qualitative results of two networks. (**a**) Input image, (**b**) label annotation, (**c**) Network1, and (**d**) Network2.

Figure 10 visualizes the segmentation results of two networks. As shown in Fig. 10, although the segmentation result of the first network was that the cell membrane class was interrupted and the accuracy was not good, the result of the final output, as shown in Fig. 10d, was better than the result using only the first network. We consider that the input image for the second network was emphasized by AEP, translated images were easier to discriminate for deep learning, and consequently, the accuracy was improved.

#### Effectiveness of AWEL

Table 6 shows the results of the ablation studies for AWEL. We evaluated our proposed method without AWEL, with AWEL using fixed weights, and with AWL using automated weights. Without AWEL, the output of the second network was only one segmented image as the final result. The fixed weights were defined as $w_i=1$. Our proposed AEP+AWEL method improved the IoU compared with only AEP and AWEL using fixed weights. The ensemble learning method that automatically determines the weights was more effective than the fixed-weight ensemble learning.

**Table 6:** Ablation study for preprocessing methods.

Methods	Mouse liver cell image dataset				iRPE cell image dataset		
	Average	Cytoplasm	Membrane	Nucleus	Average	Background	Membrane
Output	57.97±2.10	**72.85±3.80**	40.63±2.81	62.44±4.30	62.81±0.35	75.85±0.44	50.76±0.27
Output with Softmax	55.23±2.68	72.53±3.24	35.85±4.11	57.32±5.72	62.50±0.32	74.87±0.20	50.12±0.55
Output with Argmax	46.32±6.53	66.19±3.10	39.00±3.89	33.77±17.61	63.04±0.55	75.86±0.20	50.22±1.19
AEP	**59.53±1.72**	71.91±3.91	**43.27±2.32**	**63.42±3.38**	**64.05±0.45**	**75.92±0.33**	**52.19±0.75**

Significant values are in bold.

**Figure 11:**
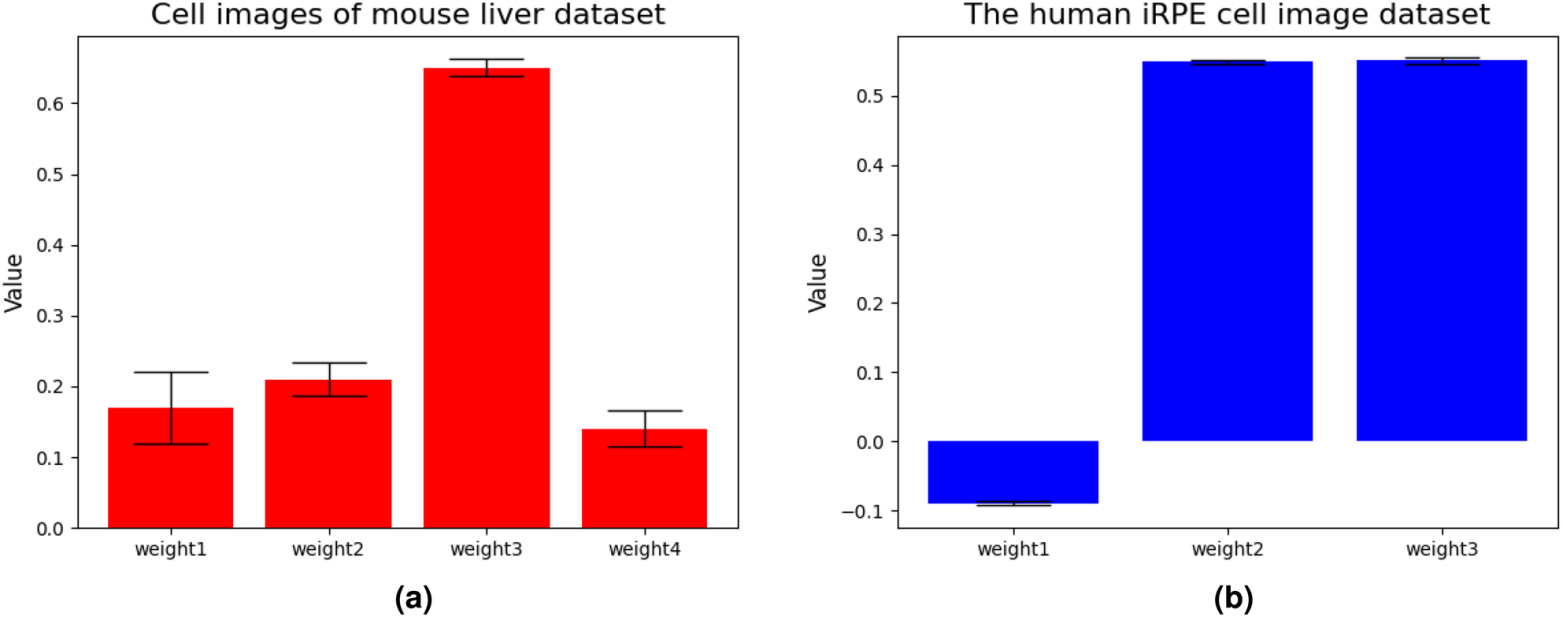
(**a**) and (**b**) are the ablations on weights used by AEP.

Figure 11a,b visualizes the results of the weights used by AWEL. We plotted the weights of the 3D convolution layer for ensemble learning using test images. The weights were the average values for cross-validation. Figure 11a shows the mouse liver cell image dataset and Fig. 11b shows the human iRPE cell image dataset. As shown in Fig. 11a, the most influential weight for ensemble learning was the third weight. This result demonstrates that the AWEL judged the translated input image corresponding to the weight3 is the most important automatically in the training stage, and it contributed to the final prediction. In Fig. 11b, although the second and third weights are the same, the first weight has a negative value. In both cases, the AWEL weights were unbiased towards a certain weight; the final segmentation results could be output using each segmentation result from the first and second networks.

#### Validation of the number of translation filters

**Figure 12:**
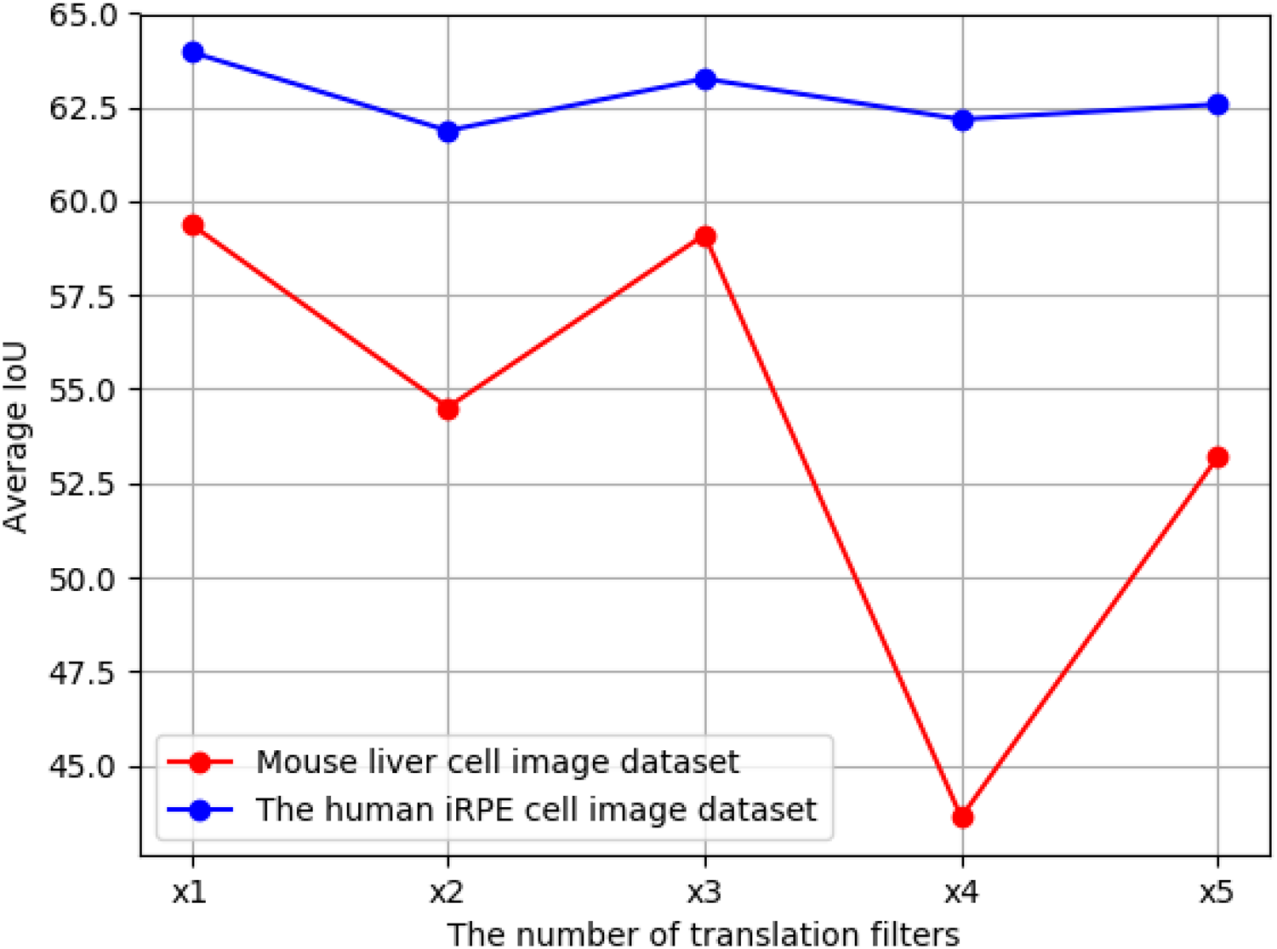
Ablation on the number of translation filters for AEP architecture. The red line is the mouse liver cell image dataset, and the blue line is the human iRPE cell image dataset.

Figure 12 shows the results of the ablation studies on the number of translation filters for AEP. We compared the number of translation filters set to double($\times 2$), triple($\times 3$), quadruple($\times 4$), and quintuple($\times 5$) the number of segmentation classes measured by the average IoU. As shown in Fig. 12, the best IoU was obtained when we set the number of translation filters to the number of classes ($\times 1$) for both cell image datasets. The average IoU tended to decrease as the number of translation filters increased. Increasing the number of translation filters is expected to result in filters that are unrelated to each object.

**Figure 13:**
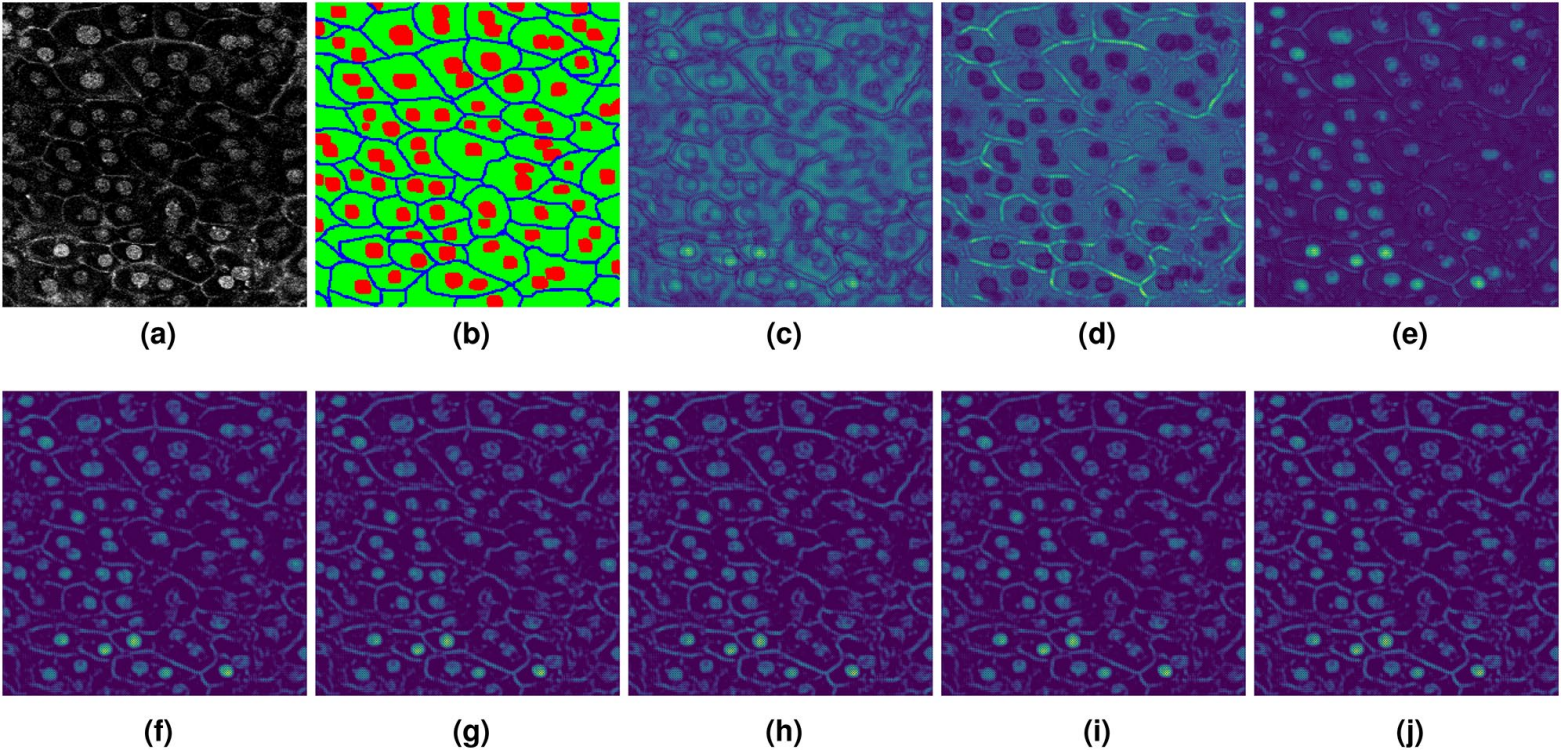
Visualization results of translation filters using AEP. (**a**) is the input image; (**b**) is the segmentation label; (**c**–**e**) are the filters when we set the number of translation filters to the number of classes ($\times 1$); and (**f**–**j**) are examples of filters when we quintupled ($\times 5$) the number of segmentation classes.

Figure 13 shows the visualization results of translation filters using AEP. As shown in Fig. 13, the generated filters were the same images when we quintupled the number of segmentation classes as translation filters ($\times 5$). Consequently, the enhancement of each class from the segmentation results was less effective. Based on this validation, we confirm that the number of translation filters should be set to the same number of segmentation classes.

## Discussion

In general, although raw cellular images tend to be low quality, all of the publicly available datasets for segmentation, which are easy to use, are quite clean and easy to train for deep learning models. Then, there are very limited of low-quality cellular image datasets for segmentation that can be used, and as a result, we only evaluated on two datasets in this study. Furthermore, to confirm the generalization performance of our proposed method, we processed publicly available clean cell image datasets to create and evaluate three types of pseudo low quality images. As shown in Table 4 and Fig. 9, our proposed method performs well even with pseudo cellular images, which we believe demonstrates the generalization performance of the proposed method.

## Conclusion

In this study, we focused on a pre-processing method for low quality cell images using deep learning, which has not been discussed, and proposed a segmentation method using automatic preprocessing and ensemble learning. In experiments on actual cell images, we translated input images into images that are easy to segment, and the average IoU improved by approximately 1.63% compared with a segmentation network without preprocessing. In addition, the proposed method performed well on another cell image dataset. From evaluation experiments using pseudo low quality cell images, we confirmed the generalization performance of our proposed method. Although our method uses the ground truth label for training the first network, by combining an unsupervised learning approach, it may be possible to add further expressiveness to the automatic preprocessing filter. This may further improve accuracy, and it is a subject for future research.

## Data Availability

Our code is available at https://github.com/usagisukisuki/AEP. The mouse liver cell image dataset generated and analyzed during the current study is not publicly available. Please request from corresponding authors^27^. The human iRPE cell image dataset generated and analyzed during the current study is available in the National Institute of Standards and Technology: https://isg.nist.gov/deepzoomweb/data/RPEimplants.
